# The Summer School of Civics

**Published:** 1920-08-28

**Authors:** 


### The Summer School of Civics.

### AN INTERESTING EXPERIMENT.

For the last four years (writes a special correspondent) the Civic Education League has endeavoured through its summer school to give wider currency to a new conception of civics. As a subject in the school curriculum civics is far from being a new department in either England or America. But as an exposition of the art of living in communities civics is still a laggard, and those who study civics are led to believe that it confines itself to local administration, or public health, or the relation of the Government to the citizen.

The recent meeting of the Summer School of Civics at High Wycombe, Bucks, did not attempt to eliminate the studies with which civics is conventionally associated, but it broadened their basis and enriched them with a new method. The courses were established with a view to appealing to three groups of students: teachers, public health, and

**THE PUBLIC WELL-BEING**—*(continued)*

other social workers, and the less specialised body of citizens from various other occupations. The programme especially designed for the first group included a course of primitive life from the psychological viewpoint, another on the foundation of civics, a third on the problem of sex, and a fourth on public administration. For the second group there was a course on the welfare of infants and young children, and for the third there was a series of lectures and discussions on the principles of reconstruction. None of these courses, it is needless to say, was in watertight compartments, and even the more specialised studies, like that on analytical psychology, had students drawn from all the groups.

The formal studies undertaken by the regular students were enriched and vivified in two ways. On the other hand, they were related to a series of lectures on reconstruction which took place in the evening throughout the fortnight. This series was opened by a brilliant and scholarly lecture by Mr. G. P. Gooch, the editor of the *Contemporary Review*, on modern ideas on the State and society, and among other items it included a survey of the field of reconstruction, by Mr. Alexander Farquharson, a study of present-day officialism, by the Chairman of the Civic Education League, Mr. Waldegrave, and a description of industrial reconstruction in the building trades, by Mr. Malcolm Sparkes. By this means the students were quickened into a sense of the immediacy and urgency of current social movements in fields with which many of them were unfamiliar. The presence of members of the local community, identified in one way or another with these movements, was a distinct advantage.

### Social Conditions from the Bottom.

The second method of intellectual enrichment was the use of the local community not merely as a place to study in, but as a convenient exemplification of the various economic and social and political processes which were described in the various courses. Each of the excursions which were taken into the surrounding region—to West Wycombe, to Beaconsfield, and to Marlow—was something more than the means of passing a recreative afternoon: they were all so many attempts to explore and understand the regional life from the ground up: that is to say, from the chalk hills of South Bucks, with their beechwood forests, up to the furniture factories, the schools, the multiple shops, and the churches, with the attendant dwelling-houses and allotment-gardens. It was this part of the course, in some respects the least formal, which proved to be the most stimulating to a large number of students who have not yet learned that the social sciences can be studied by direct observation in the same fashion that botany or geology are best pursued. The regional surveys included a walk through the commercial and financial centre of High Wycombe (parallel in so many ways, albeit on diminished scale, to great London itself), the inspection of paper mills and furniture-making factories, and the examination of various rudimentary upland villages.

The success of this experiment in regional survey and in the use of a small industrial community, in close contact with its rural environs, as the centre of a summer school is well attested by a vote taken in one of the classes, where the majority of the students favoured the selection of a similar town for the next meeting, and where by a unanimous vote they rejected the suggestion to choose a seaside town, such as would be especially adapted to recreation and residence. It is too early to say what the effects of the summer school will be upon the local community, but the agreement of various leading citizens on the fact that the summer school has served as helpful stimulus to improvement in High Wycombe can hardly be ignored. It may well prove that an itinerant summer school of civics, conducted on the lines that have been so successfully laid down in this High Wycombe experiment, promises rewards to the community in which it temporarily settles even greater than those which the students themselves enjoy.

